# Antrodia Camphorata Polysaccharide activates autophagy and regulates NLRP3 degradation to improve liver injury-related inflammatory response

**DOI:** 10.18632/aging.204330

**Published:** 2022-10-10

**Authors:** Shuiliang Ruan, Yi Yang, Wenyan Li

**Affiliations:** 1 Department of Gastroenterology, The Second Affiliated Hospital of Jiaxing University, Jiaxing 314001, Zhejiang Province, China; 2 Department of Pharmacy, The Second Affiliated Hospital of Jiaxing University, Jiaxing 314001, Zhejiang Province, China

**Keywords:** autophagy, Antrodia Camphorata Polysaccharide, liver injury, Kupffer cells, inflammatory response

## Abstract

This study illustrated the liver protection mechanism of ACP from the perspective of autophagy activation. ACP suppressed the inflammatory injury of KCs, and decreased the cell apoptosis rate. After LTG and LC3 staining, ACP promoted lysosomal production, increased LC3 expression, activated autophagy, and suppressed the expression of NLRP3 and inflammatory factors. Under the electron microscope, ACP accelerated the production of autophagosomes. After simultaneous treatment with 3-MA and ACP, the effect of ACP on resisting KC injury decreased, the expression of NLRP3 was up-regulated, and autophagy was suppressed. As discovered in the mouse model of liver injury, ACP inhibited the ALT and AST levels, promoted the occurrence of autophagy, reduced NLRP3 expression and alleviated liver injury.

ACP activates autophagy to induce NLRP3 degradation, thus suppressing inflammatory response in liver injury and exerting the liver protection effect, which is one of the mechanisms of action of ACP.

## INTRODUCTION

At present, the incidence of liver injury shows an increasing trend year by year, and it involves several pathological types, like drug-induced injury, alcoholic liver injury and immune injury commonly seen in clinic [1]. Inflammatory response is considered as the major pathological factor for liver injury. More and more studies have discovered that NLRP3 inflammasome is closely related to liver injury, and abnormal NLRP3 up-regulation is discovered in multiple liver injury models [2], especially in Kupffer cells (KCs) [3]. NLRP3 inflammasome is also one of the media for the maturation and release of multiple inflammatory factors. How to reduce NLRP3 formation is the key to suppressing liver injury-related inflammation. Antrodia Camphorata (AC) is a fungus species belonging to Polypores unique in Taiwan. Through component analysis of AC, we discover that the content of Antrodia Camphorata Polysaccharide (ACP) in AC is significantly higher than those in fungi of the same class, and is of great research and development value [4, 5]. In local Taiwan, AC is mainly used to treat liver injury, alcoholic liver, non-alcoholic fatty liver disease (NAFLD) and liver cancer, and attains favorable therapeutic effect. But its precise material basis and pharmacodynamic effect have not been illustrated yet.

Autophagy is a lysosome activation-dependent degradation pathway, which is important for regulating protein and nucleic acid degradation. It is found in liver injury that, the autophagy level was up-regulated in acute phase, which exerts the protective effect [6, 7], but the role of autophagy activation in inflammatory response and the effect of NLRP3 remain unclear. Therefore, this study aimed to reveal the role and mechanism of ACP in resisting liver injury.

## MATERIALS AND METHODS

### Cell culture

The mouse KCs (Wuhan Procell Biotechnology Co., Ltd, Wuhan, China) were cultured in the RMPI 1640 complete medium supplemented with 10% FBS in the incubator under 37° C and 5% CO_2_ conditions. After cell passage, the cell viability was tested by trypan blue reagent, and KCs at logarithmic phase were harvested for experiments. KCs were then divided into DMSO (control), LPS and ACP groups. Cells in LPS group were treated with 1 mg/L LPS to induce inflammatory response, while those in ACP group were pretreated with high-dose (15 mg/L) and low-dose (5 mg/L) ACP for 6 h, and then exposed to LPS to induce inflammatory response.

In the assay to verify the mechanism of ACP through activating autophagy, KCs were divided into DMSO, LPS, ACP and 3-MA+ACP groups. Cells in ACP group were treated with 15 mg/L ACP, those in 3-MA+ACP group were pretreated with the autophagy inhibitor 3-MA (25 μM) to suppress autophagy and intervened with 15 mg/L ACP for 6 h before experiments.

### CCK-8

Cells were inoculated into the 96-well plates, the serum-free medium was changed after cell adherence, and LPS was then added to induce for 12 h. Later, each well was added with 10 μl CCK-8 solution to incubate for 4 h, and the absorbance (OD) value was detected at 450 nm. Meanwhile, the blank medium was set for background absorption correction.

### Flow cytometry (FCM)

Cells were inoculated into the 6-well plates and grouped after adherence according to the aforementioned method. After LPS induction for 12 h, the adherent and suspension cells were collected, washed with pre-chilled PBS twice, and centrifuged at 3000 rpm/min for 30 min. After suspension with Binding Buffer, cells were stained with 5 μl Annexin V-FITC for 5 min in dark and then with 5 μl PI in dark for another 5 min. After washing with PBS, cells were loaded for test using the cell apoptosis detection kit (BD, MA, USA).

### Enzyme-linked immunosorbent assay (ELISA)

The levels of inflammatory factors IL-1β, IL-18 and TNF-α in the medium were determined in cell assays. In brief, cells were inoculated into the 12-well plates and cultured with serum-free medium after adherence. After LPS induction for 12 h, the cell medium was collected. Afterwards, the suspension cells and cell debris were removed, and the supernatant was collected to detect the inflammatory factor levels by the standard curve method in line with the ELISA kit (Nanjing Institute of Biological Engineering, Nanjing, China) instructions. The results were expressed as ng/ml.

Detection of inflammatory factors in tissues and serum. The peripheral blood samples were centrifuged to collect the upper layer serum, meanwhile, liver tissues were grinded with liquid nitrogen, lysed with 1.0 ml NP-40 lysate on ice for 30 min and the supernatant was collected for protein quantification according to the methods in cell experiments.

### Immunofluorescence (IF) staining

The levels of LC3 and NLRP3 in cells were detected by IF staining. Briefly, the sterile slides were added into the 6-well plates. After LPS treatment, KCs were fixed with 4% paraformaldehyde (PFA), permeabilized with 0.2% Triton X-100, blocked with 2% BSA, and incubated with monoclonal antibodies against LC3 and NLRP3 (dilution, 1:200-300) under room temperature. Later, cells were labeled and incubated with the fluorescence-labeled IgG antibody (Abcam, MA, USA). In NLRP3 staining, the cell nuclei were stained with DAPI. After washing twice with PBS, DAPI was added and cells were observed under the fluorescence microscope.

### Lysosome probe LTG staining [8]

After adherence, KCs were treated with LPS for 6 h, incubated with 200 μl lysosome-labeled probe (LTG, 50 nmol/L) for 30 min, and washed with fresh medium thrice. Then, PBS was added dropwise to cover the cells, which showed green fluorescence after lysosome staining under the fluorescence microscope.

### Autophagy observed under the electron microscope

After adherence, KCs were treated with LPS for 6 h, fixed with 2.5% glutaraldehyde, dehydrated with ethanol, and embedded with Epon812 epoxy. Afterwards, the sample was sliced into ultra-thin sections using the LKB III type ultra microtome. Then, sections were double-stained with uranyl acetate and lead citrate, and the changes in ultrastructure were observed with the EM420 transmission electron microscope.

### Western-blot (WB) assay

KCs were collected after LPS induction for 12 h. washed and lysed with 1.0 ml NP-40 lysate (Beyotime Biotechnology Co., Ltd, Shanghai, China) for 30 min on ice. Then, the mixture was diluted with 5x loading buffer to 20 μl, and boiled to denature the proteins. Afterwards, proteins were separated by SDS-PAGE, and transferred onto the PVDF membranes. Membranes were then blocked with 5% skimmed milk powder, then incubated with TBST-diluted monoclonal antibodies against LC3, P6 and NLRP3 overnight at 4° C, and later with HRP-IgG (Abcam, MA, USA). Finally, the sections were detected by chemiluminiscence, the OD was analyzed using Image Pro-Plus 6.0 software, and the results were expressed as the OD ratio of target protein to GAPDH.

To detect proteins in tissues, proteins were extracted and detected according to the methods in ELISA.

### Construction of the mouse model of liver injury [9]

The SPF C57BL/6 mice were randomly divided into Control, D-GalN/LPS (D/L) and ACP groups. Mice in ACP group were given intragastric administration of ACP at 5 mg/kg and 15 mg/kg once a day for 7 consecutive days. While mice in Control and D/L groups were given intragastric administration of equivalent volume of normal saline once a day. At 24 h after the last administration, mice in D/L and ACP groups were intraperitoneally injected with 1000 mg/kg D-GalN (Sigma, MA, USA) and 10 μg/kg LPS (Sigma, MA, USA) to construct the acute liver injury model.

### Detection of ALT and AST

The ALT and AST expression was detected using corresponding kits. In brief, after LPS/D-GalN intervention for 48 h, the tail venous blood was collected from each mouse, and centrifuged to collect the supernatant to detect ALT and AST levels by ultraviolet colorimetry (Nanjing Institute of Biological Engineering, Nanjing, China) according to the kit instructions. The AST and ALT results were expressed as U/L.

### H&E staining

At 48 h after LPS/D-GalN injection, mice were sacrificed through carbon dioxide suffocation. Then, the liver tissues were dissected, embedded in paraffin and prepared into the 4 μm consecutive sections. The detailed steps were shown below, xylene deparaffinage, gradient dehydration with 100%, 95% and 80% ethanol, washing with tap water for 2 min, staining with hematoxylin for 3 min, washing with tap water for 2 min, treatment with 1% hydrochloric acid alcohol for 2 s, washing by tap water for 2 min, and treatment with 1% ammonia water for 20 s, followed by 10 s staining with 0.5% eosin ethanol, gradient dehydration of ethanol, xylene transparentizing, and neutral balsam mounting. Finally, the liver tissue pathological changes were observed under the light microscope.

### Immunohistochemical (IHC) staining

To detect NLRP3 expression in tissues, the paraffin-embedded liver tissue block was sliced into 4 μm serial sections, deparaffinized in xylene, and treated with gradient ethanol, followed by antigen retrieval. The endogenous peroxidase was eliminated by 3% hydrogen peroxide. Later, sections were blocked with 2% bovine serum albumin (BSA) at 37° C. After non-specific antigen-antibody binding, sections were incubated with anti-NLRP3 monoclonal antibody (dilution, 1:250; Abcam, MA, USA) and subsequently with peroxidase-labeled streptomycin (Abcam, MA, USA) for 15 min. Afterwards, each section was added with drops of freshly prepared DAB color developing solution (DAKO, Denmark), sufficiently washed with tap water, countered-stained with hematoxylin, and mounted.

### Statistical analysis

Statistical analysis was completed using SPSS19.0 software. The measurement data were expressed as mean± standard deviation $(\bar{x}\pm \text{s)}$. One-way ANOVA was adopted for comparison among multiple groups, whereas SNK test was utilized for comparison between groups. P<0.05 stood for statistical significance.

### Availability of data and material

The data and material were availability.

## RESULTS

### ACP activated autophagy and promoted NLRP3 degradation to protect KCs against inflammatory injury

**Figure 1:**
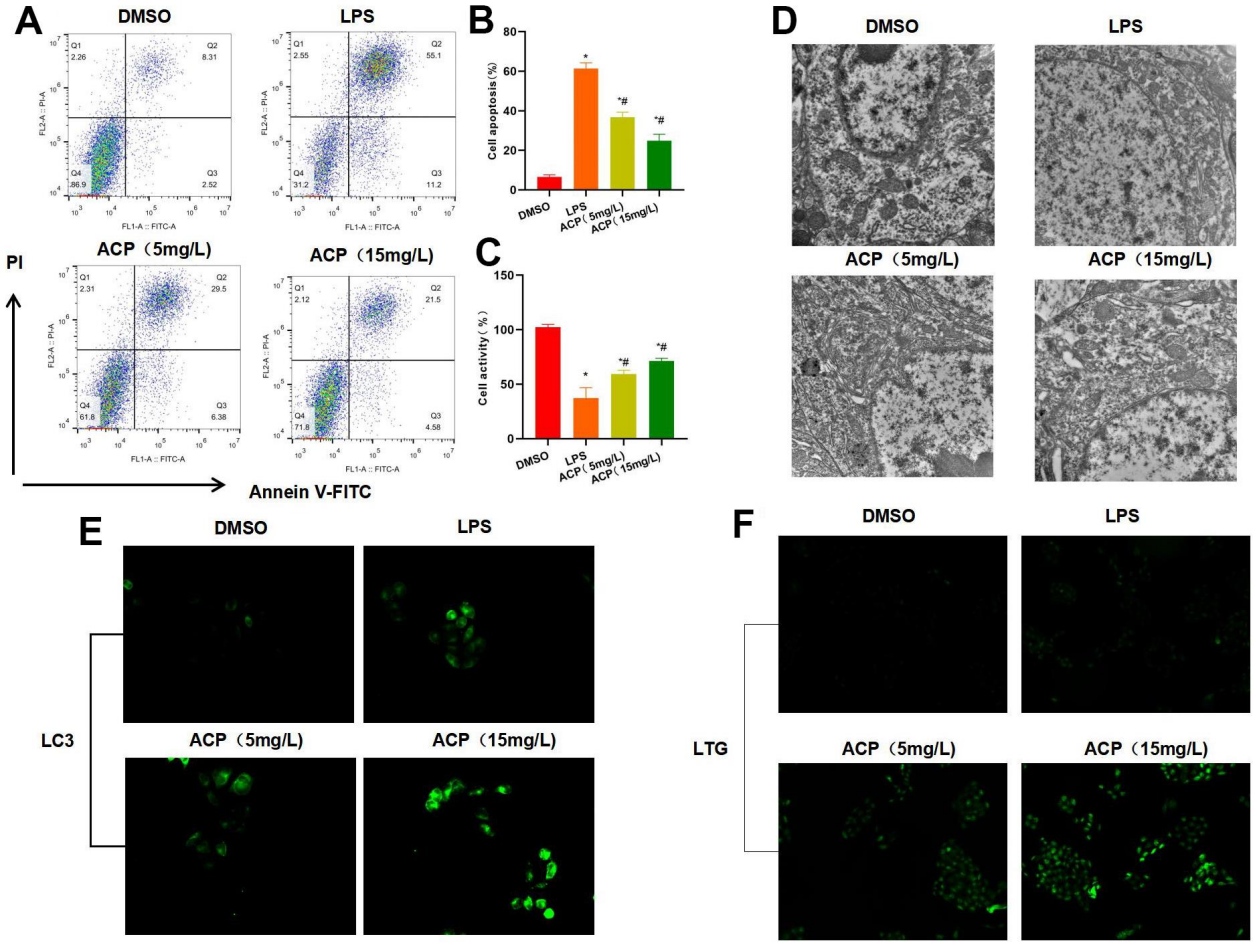
**ACP suppressed cell injury and activated autophagy (n=3).** (**A**, **B**) Flow cytometry revealed that LPS induced cell injury and significantly increased the apoptosis rate, while ACP suppressed cell apoptosis in a dose-dependent manner. ^*^P<0.05 compared with DMSO, ^#^P<0.05 compared with LPS. (**C**) Cell viability test suggested that cell viability decreased in LPS group, lower than that of DMSO group, while ACP suppressed LPS. ^*^P<0.05 compared with DMSO, ^#^P<0.05 compared with LPS. (**D**) Electron microscope observation demonstrated no obvious lysosome or autophagosome formation in DSMO, while weak autophagy activation was seen in LPS group, and obvious autophagosomes were detected in ACP, suggesting that ACP activated autophagy. (**E**) LC3 fluorescence staining revealed that LC3 was not significantly activated in DMSO, lowly expressed in LPS and significantly up-regulated in ACP, higher than that in DMSO and LPS group. (**F**) Lysosome probe LTG analysis suggested that ACP promoted lysosome formation and markedly enhanced the fluorescence intensity.

LPS induced KC injury, and the cell apoptosis rate significantly increased, higher than that of DMSO group. While ACP suppressed cell apoptosis in a dose-dependent manner, and the cell apoptosis rate in ACP group was significantly lower than that in LPS group (Figure 1A, 1B). Cell viability test also suggested that, LPS suppressed cell viability, which was lower than DMSO group, while the cell viability in ACP group was higher than LPS group, and high-dose ACP had superior effect to low-dose ACP (Figure 1C). The formation of lysosomes and autophagosomes was observed under electron microscope, as a result, DMSO group did not exhibit any obvious lysosome or autophagosome formation, while weak autophagy activation was seen in LPS group, and obvious autophagosomes were observed in ACP group, suggesting that ACP activated the autophagy process (Figure 1D). LC3 staining results suggested that, LC3 was not obviously activated in DMSO group, while it was weakly expressed in LPS group, and significantly up-regulated in ACP group. LC3 is the key marker protein of autophagy, suggesting that ACP activated autophagy (Figure 1E). Lysosome LTG probe staining assay also indicated that ACP promoted lysosome formation, and the fluorescence intensity was markedly enhanced (Figure 1F).

**Figure 2:**
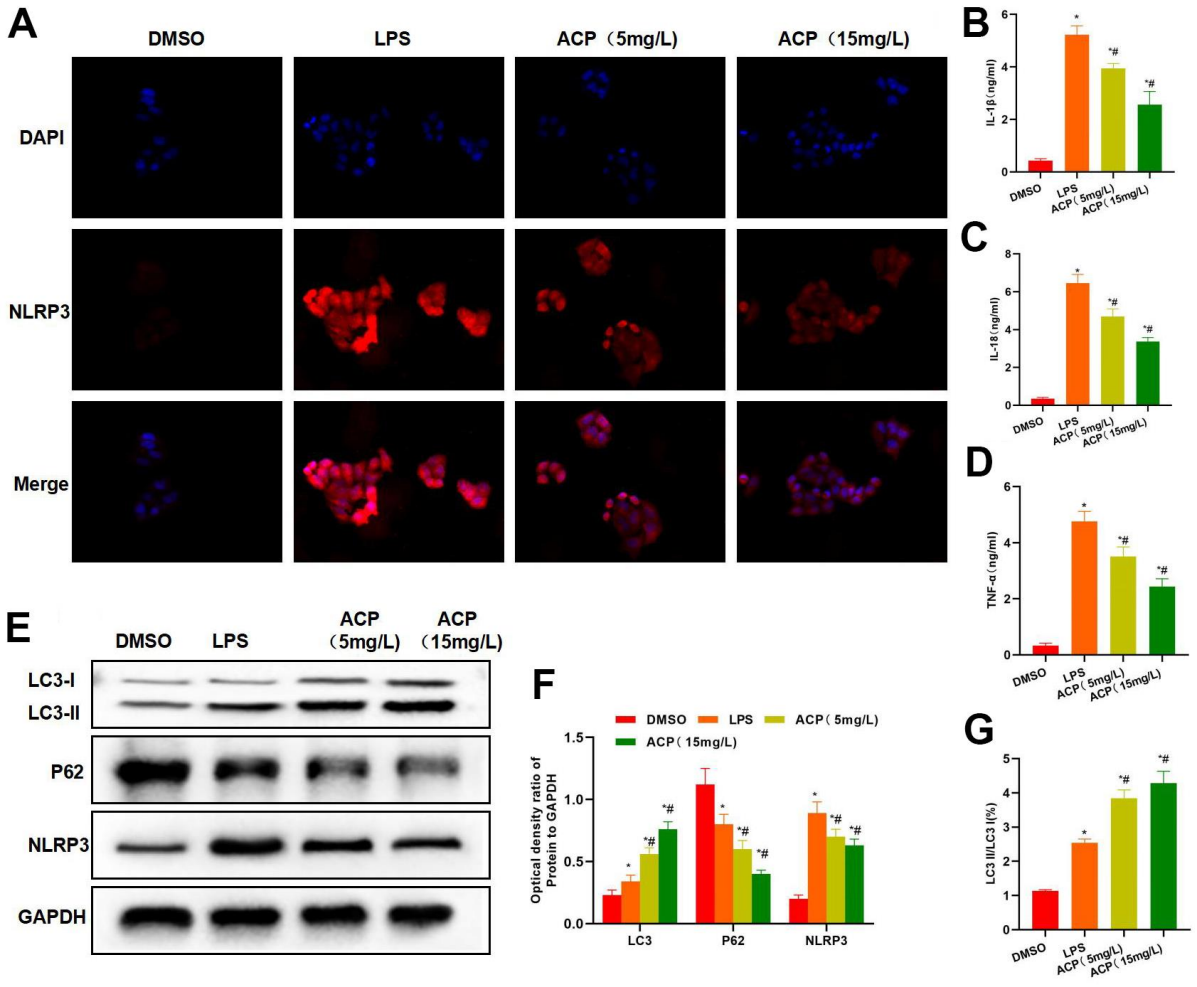
**ACP suppressed inflammatory response and NLRP3 activation.** (**A**) NLRP3 fluorescence staining revealed that, LPS promoted NLRP3 expression, and the fluorescence intensity significantly increased, while ACP suppressed NLRP3 expression, significantly lower than LPS group. (**B**–**D**) Inflammatory factor detection revealed that, ACP decreased the expression of IL-1β, IL-18 and TNF-α, and the difference was significant compared with LPS group. ^*^P<0.05 compared with DMSO, ^#^P<0.05 compared with LPS. (**E**, **F**) Protein detection indicated that LC3 expression significantly increased in ACP group, P62 and NLRP3 expression decreased, and NLRP3 expression was negatively correlated with LC3 expression. At the same time, LC3 II expression increased, showing that autophagy was activated. ^*^P<0.05 compared with DMSO, ^#^P<0.05 compared with LPS. (**G**) Ratio of LC3 I to LC3 II significantly increased when LC3 I was transformed into LC3 II, suggesting that autophagy was activated and the autophagy flow was smooth. ^*^P<0.05 compared with DMSO, ^#^P<0.05 compared with LPS.

IF staining results also indicated that NLRP3 was up-regulated in LPS group, and the difference was significant compared with DMSO group, while NLRP3 expression was down-regulated in ACP in a dose-dependent manner (Figure 2A). In inflammatory factor detection, ACP decreased the expression of IL-1β, IL-18 and TNF-α, and the differences were significant relative to LPS group (Figure 2B–2D). Protein detection results indicated that LC3 expression evidently elevated in ACP group, meanwhile, P62 and NLRP3 expression decreased, and NLRP3 expression was negatively correlated with LC3 expression. Meanwhile, the expression of LC3II increased, suggesting that autophagy was activated (Figure 2E, 2F). The ratio of LC3 I to LC3 II also demonstrated that ACP promoted autophagy, and LC3 I was transformed into LC3 II (Figure 2G).

### Suppressing autophagy antagonized the effect of ACP, and promoted inflammatory response and injury of KCs

**Figure 3:**
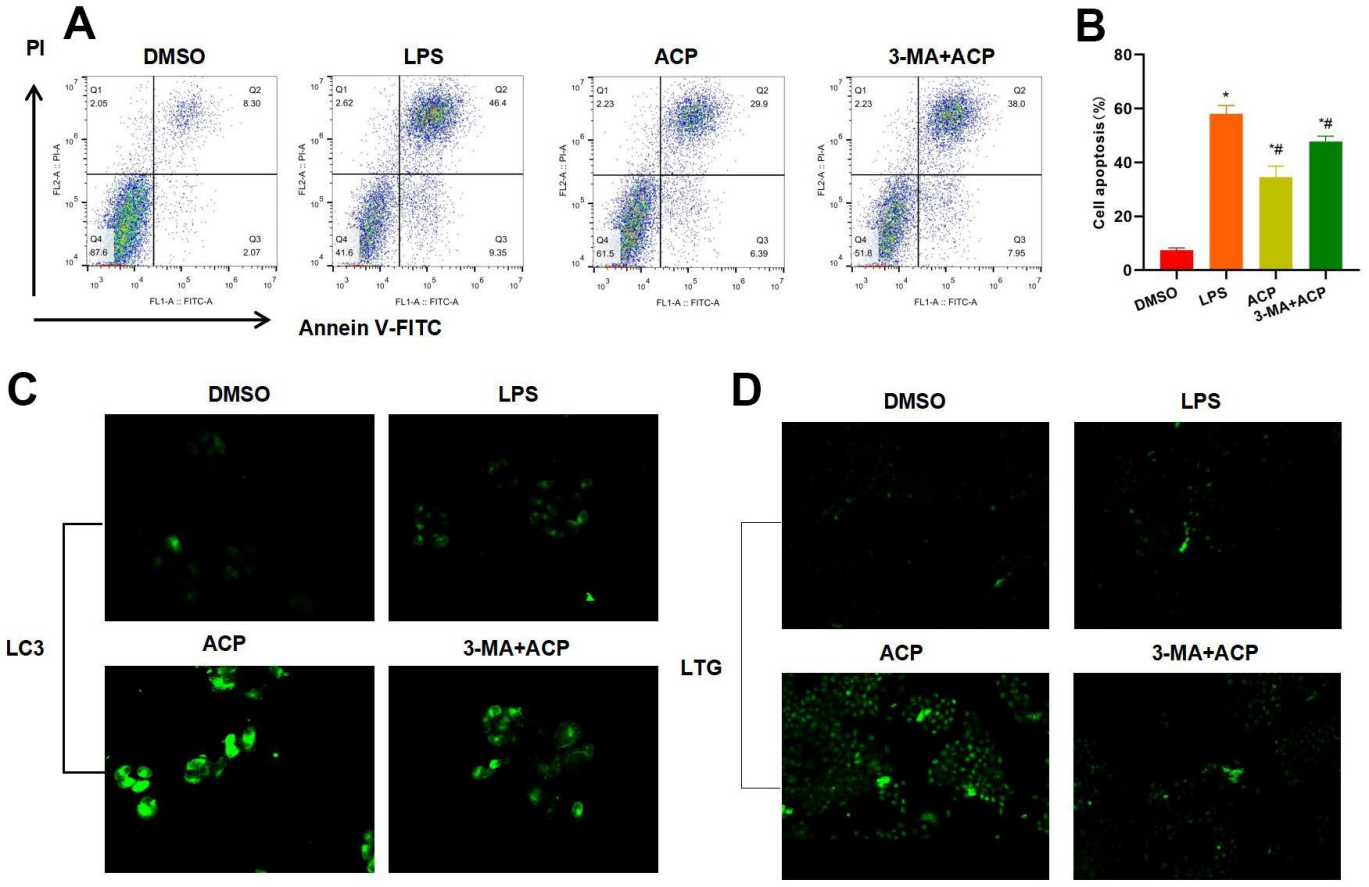
**Suppressing autophagy antagonized the effect of ACP (n=3).** (**A**, **B**) Flow cytometry results revealed that 3-MA suppressed autophagy, and the effect of ACP was antagonized. Moreover, the cell apoptosis rate in 3-MA+ACP group significantly increased, higher than that of ACP group. ^*^P<0.05 compared with DMSO, ^#^P<0.05 compared with LPS. (**C**) LC3 staining suggested that 3-MA suppressed LC3 expression, and the fluorescence intensity was weakened. (**D**) LTG probe detection suggested that 3-MA suppressed lysosome formation.

Autophagy was suppressed by the autophagy inhibitor 3-MA. The results indicated that the effect of high-dose ACP was antagonized. FCM results indicated that the cell apoptosis rate in 3-MA+ACP group significantly increased, higher than that of ACP group, which suggested that suppressing autophagy antagonized the effect of ACP (Figure 3A, 3B). Moreover, 3-MA suppressed LC3 expression, and the LC3 staining fluorescence intensity significantly decreased in 3-MA+ACP group, lower than that in ACP group. In the meantime, LTG probe detection revealed the weakened lysosome formation in 3-MA+ACP (Figure 3C, 3D).

**Figure 4:**
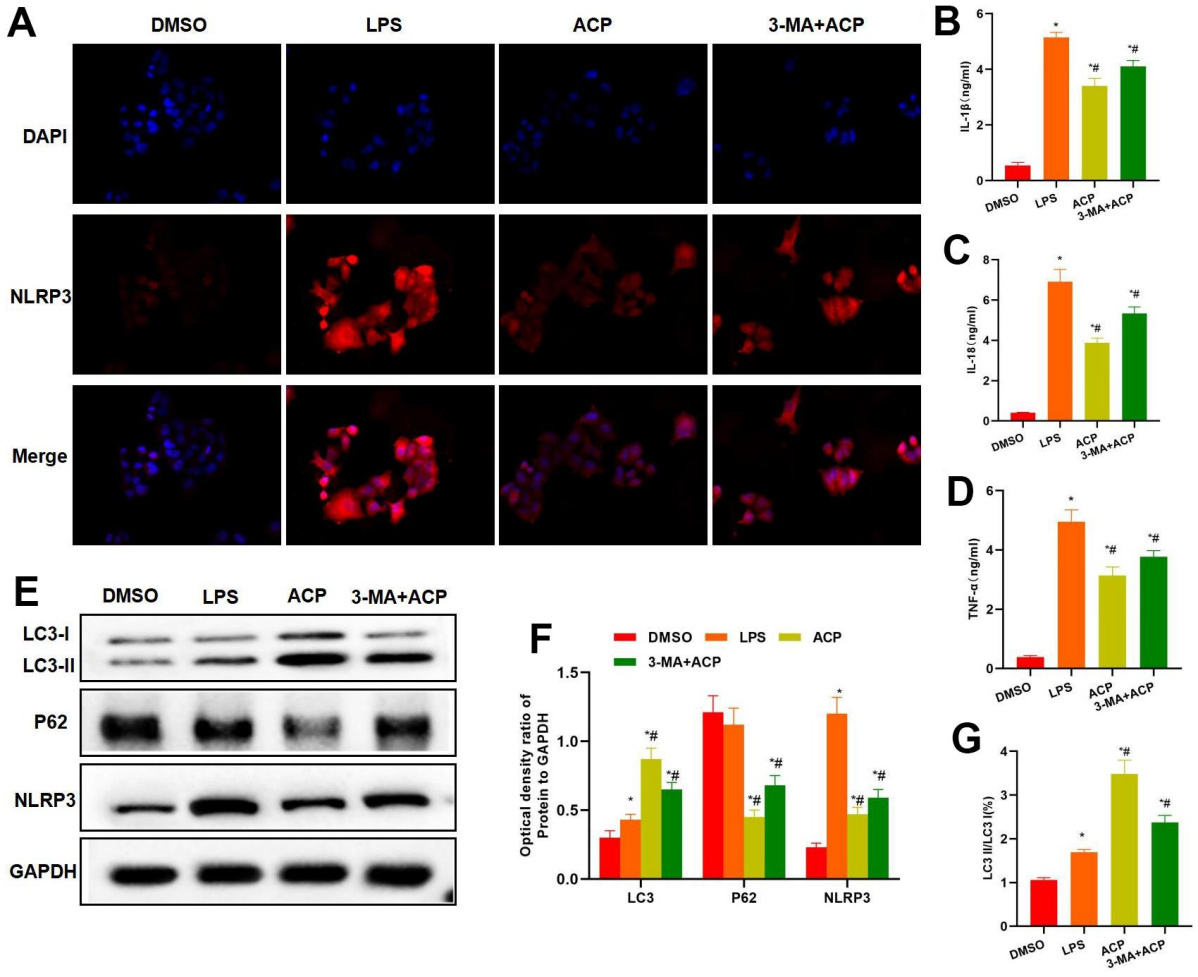
**Suppressing autophagy enhanced inflammatory response and NLRP3 expression.** (**A**) NLRP3 staining results indicated that, 3-MA antagonized the effect of ACP, and NLRP3 expression markedly increased in 3-MA+ACP group, higher than that in ACP group. (**B**–**D**) The expression levels of inflammatory factors IL-1β, IL-18 and TNF-α in 3-MA+ACP group were higher than those in ACP group. ^*^P<0.05 compared with DMSO, ^#^P<0.05 compared with LPS. (**E**–**G**) Protein detection demonstrated that 3-MA suppressed autophagy, LC3 expression decreased in 3-MA+ACP group, while NLRP3 and P62 expression increased, and the differences were significant compared with ACP group. Meanwhile, the LC3 II/LC3 I ratio decreased. ^*^P<0.05 compared with DMSO, ^#^P<0.05 compared with LPS.

According to the results of NLRP3 staining, 3-MA antagonized the effect of ACP, NLRP3 expression was significantly up-regulated in 3-MA+ACP group, higher than that of ACP group (Figure 4A). Inflammatory factor detection revealed that the expression levels of inflammatory factors IL-1β, IL-18 and TNF-α in 3-MA+ACP group were higher than those in ACP group (Figure 4B–4D). Protein detection discovered that, 3-MA suppressed the effect of ACP and inhibited the occurrence of autophagy. LC3 expression decreased in 3-MA+ACP group, while NLRP3 and P62 expression increased, and the differences were significant compared with ACP group. Meanwhile, the LC3 II/LC3 I ratio decreased, indicating that autophagy was suppressed (Figure 3E–3G).

### ACP suppressed liver injury and inflammatory response in mice

**Figure 5:**
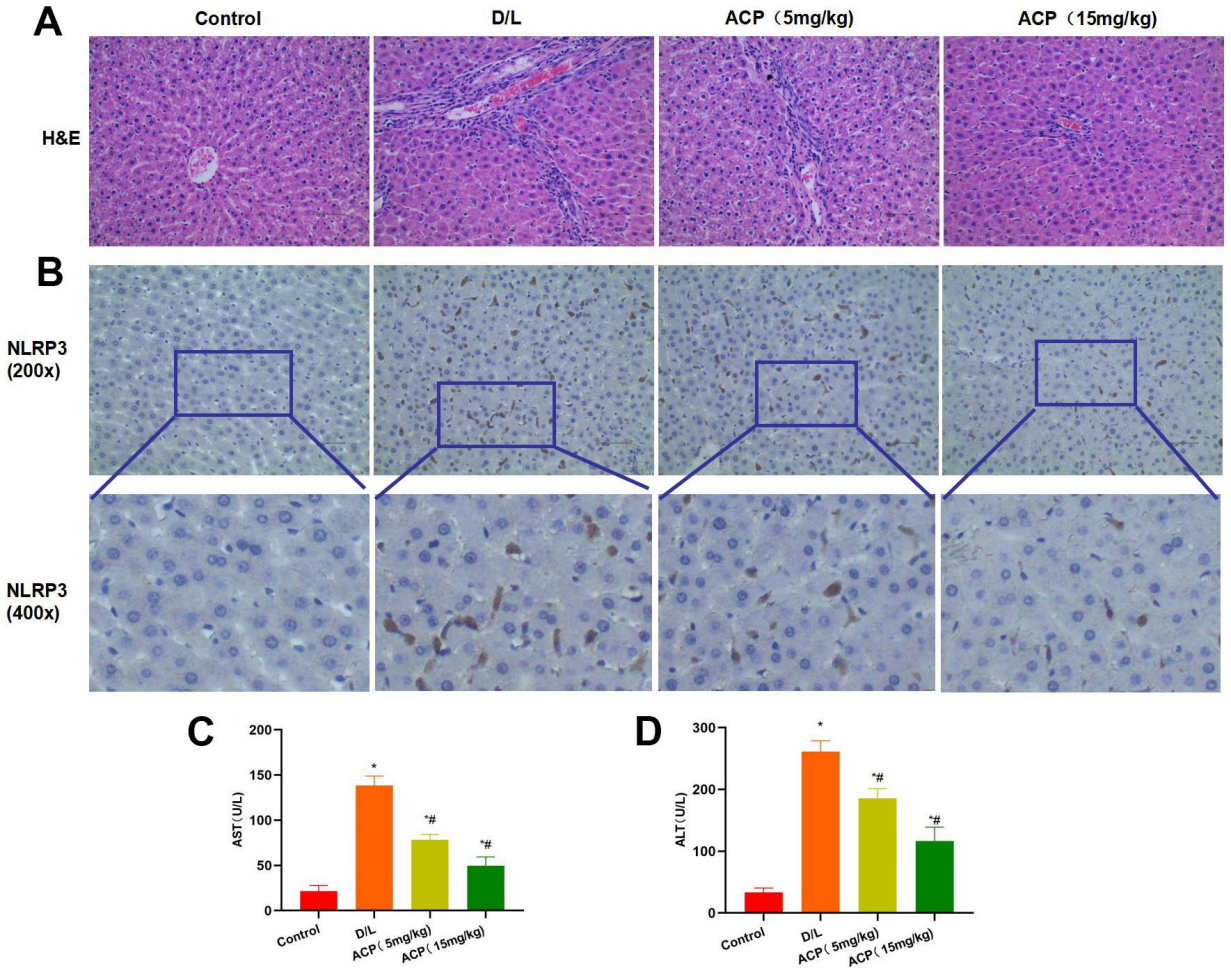
**ACP suppressed liver injury in mice (n=5).** (**A**) In H&E staining, obvious inflammatory response and injury were observed in liver tissues of D/L group, along with distinct bubble-like lesions, while ACP suppressed tissue inflammation and alleviated injury. (**B**) In NLRP3 staining, NLRP3 expression significantly increased in D/L group, while it was not expressed in Control group, demonstrating that liver injury led to NLRP3 activation. ACP dramatically reduced NLRP3 expression in tissues, and the difference was significant compared with D/L group. (**C**, **D**) In ALT and AST detection, ACP alleviated liver injury, and reduced ALT and AST expression in a dose-dependent manner. ^*^P<0.05 compared with Control, ^#^P<0.05 compared with D/L.

D-GalN and LPS induced acute liver injury in mice. After H&E staining, mice in D/L group exhibited prominent inflammatory response and tissue bubble-like lesions, with obvious liver injury. ACP alleviated tissue inflammatory response and injury, and the difference was significant compared with D/L group (Figure 5A). NLRP3 expression was significantly up-regulated in D/L group, while it was not expressed in Control group, suggesting that liver injury resulted in the activation of NLRP3. ACP significantly reduced NLRP3 expression in tissues, and the difference was significant compared with D/L group (Figure 5B). The detection results of AST and ALT indicated that, ACP mitigated the liver injury level, and decreased ALT and AST expression in a dose-dependent manner (Figure 5C, 5D).

**Figure 6:**
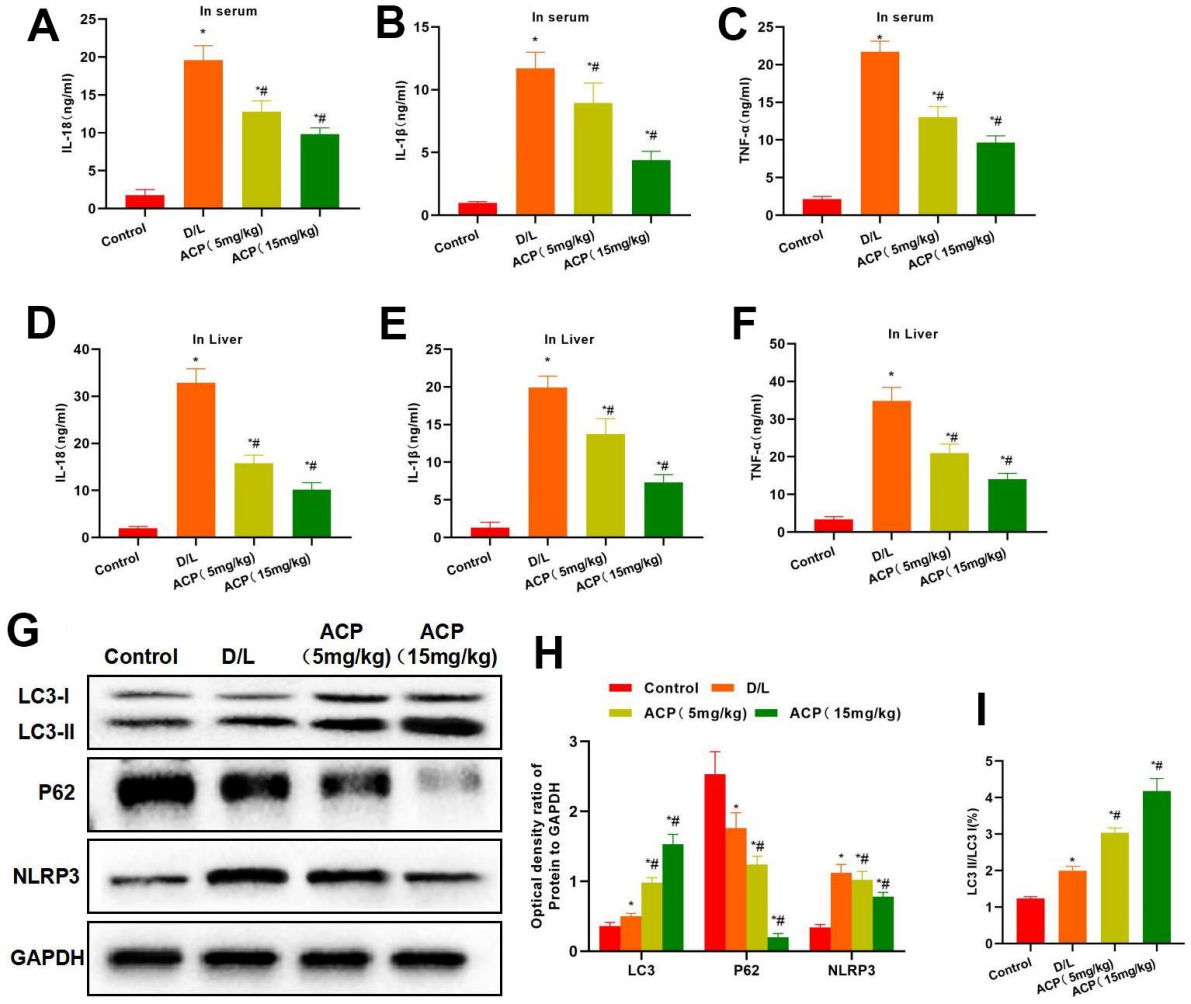
**ACP suppressed inflammatory response and activated autophagy.** (**A**–**F**) As discovered from serum and tissue inflammatory factor detection, the expression of serum and tissue inflammatory factors in D/L group increased, which was significantly higher than that of Control group. While ACP reduced the serum inflammatory factor levels in a dose-dependent manner. ^*^P<0.05 compared with Control, ^#^P<0.05 compared with D/L. (**G**–**I**) In protein detection, ACP promoted LC3 expression in tissues while suppressing NLRP3 expression, meanwhile, P62 expression also increased, and the LC3 II/LC3 I ratio increased in tissues. ^*^P<0.05 compared with Control, ^#^P<0.05 compared with D/L.

According to the detection results of serum inflammatory factors, the serum expression levels of inflammatory factors increased in D/L group, which were significantly higher than those in Control group. While ACP reduced serum levels of inflammatory factors (Figure 6A, 6B). The inflammatory factor levels in liver tissues of D/L group were substantially up-regulated, remarkably higher than those in Control group. ACP evidently reduced the inflammatory factor levels in tissues in a dose-dependent manner (Figure 6D, 6E). In protein detection, ACP promoted LC3 expression in tissues and suppressed NLRP3 expression, meanwhile, the LC3 II/LC3 I ratio in tissues increased, proving that ACP promoted autophagy (Figure 6G–6I).

## DISCUSSION

Inflammasome is a kind of multi-protein complex located in cells, which is involved in the inflammation and immune responses in human body [9]. NLRP3 inflammasome is the NOD-like receptor complex that has been most extensively investigated currently [10]. NLRP3 is mainly expressed in dendritic cells (DCs), mononuclear cells and macrophages [11]. Under physiological condition, NLRP3 is almost not expressed in primary hepatocytes [12], but stimulation by LPS or chemical substances can induce the high expression of NLRP3 [13]. The activated inflammasome can cleave the inactive procaspase-1 into active caspase-1, while the active caspase-1 can cleave pro-IL-1β and pro-IL-18 into the active IL-1β and IL-18 to amplify the inflammatory response and mediate inflammatory cell death [14]. NLRP3 is the core of inflammatory response [15]. More and more research results suggest that, NLRP3 inflammasome is related to acute liver injury. After drugs like carbon tetrachloride are injected to induce liver injury, the proportions of immune cells like neutrophils and mononuclear cells in liver tissues increase, and NLRP3 expression elevates, especially in KCs and hepatic sinusoidal endothelial cells (HSECs) [16]. As discovered from *in-vitro* experiment, after LPS stimulation, NLRP3 expression significantly increases in HSCs and hepatocytes, along with higher levels of IL-1β, IL-18 and TNF-α [17]. Moreover, it is found in peripheral blood mononuclear cell experiment in patients with acute liver failure (ALF) that, LPS stimulation increases IL-1β expression [18]. Therefore, NLRP3 has become an intervention target in liver injury research. How to suppress NLRP3 activation and inflammatory factor release is our research focus.

Autophagy also exerts an important role in regulating the inflammatory response process. Numerous experimental studies have discovered that there is negative regulatory relation between autophagy and inflammasome [19]. As found from research of autophagy negatively regulating NLRP3, the NLRP3 inflammasome activation is enhanced in cells with autophagy defect. Some research indicates that [20], the autophagy function is weakened during the aging process, which promotes NLRP3 activation. Razani et al. discovered that, during the atherosclerosis process, autophagy suppression also promoted NLRP3 activation, suggesting that autophagy negatively regulated NLRP3 activation. Autophagosome can directly wrap and degrade the inflammasome components, and the co-existence of NLRP3 and ASC after GFP-LC3/LAMP-1 labeling indicates the degradation of inflammasome in autophagosome. It is also reported that [21], autophagy inhibitor 3-MA also increases the activation of inflammasome, and induces the secretion of IL-1β and IL-18; besides, in macrophages with Atg16L1 and Atg7 knockdown, LSP stimulation increases the secretion of IL-1β and IL-18 [22, 23]. AC is a kind of natural fungus belonging to Polyporales. Existing research discovers that AC shows favorable liver protective effect, and polysaccharide is one of the abundant components in AC. This work aimed to illustrate the mechanism of action of ACP from the anti-inflammation perspective. In this study, we discovered that LPS induced NLRP3 activation in KCs and the release of massive inflammatory factors, highly consistent with literature reports. After ACP intervention, we discovered that NLRP3 expression was significantly down-regulated in KCs. Noteworthily, ACP intervention activated the autophagy process, obvious autophagosomes and vesicle structures were formed in KCs, and LC3 expression increased. LC3 is the key regulatory protein of autophagy [24]. According to our results, LC3 I was transformed into LC3 II, the ratio increased, and P62 expression decreased. P62 is the substrate protein of autophagy [25], autophagosome can be formed after P62 degradation, and with the activation of autophagy, NLRP3 expression significantly decreased. We suggested that ACP activated autophagy to induce NLRP3 degradation. To prove our conclusion, we further treated cells with the autophagy inhibitor 3-MA [26]. After simultaneous ACP+3-MA treatment, the effect of ACP was significantly weakened. When autophagy was suppressed, ACP failed to achieve the expected effect, cell injury was obvious, the secretion of inflammatory factors was up-regulated, and NLRP3 expression also increased. Therefore, we verified that ACP activated autophagy to result in NLRP3 degradation. In animal model research, we also discovered that ACP suppressed acute liver injury in mice. To be specific, ACP restrained inflammatory response and bubble-like lesions in liver tissues, and dramatically decreased the expression of inflammatory factors in serum and tissues. We also measured the expression of autophagy-related proteins in tissues. As a result, ACP promoted LC3 expression, decreased P62 and NLRP3 expression, increased LC3 II expression, and accelerated the transformation of LC3 I to LC3 II, demonstrating the smooth autophagy flow.

## CONCLUSIONS

Collectively, results in this work suggest that ACP activates autophagy and induces NLRP3 degradation in KCs to alleviate inflammatory response and suppress inflammatory factor release. ACP activates autophagy and reduces NLRP3 expression in liver injury to exert its liver protective effect, which is one of the mechanisms of action of ACP.
